# Evaluation of radiographic quality of root canal obturation performed by undergraduate and postgraduate clinical dental students in a Nigerian hospital

**DOI:** 10.11604/pamj.2024.47.166.39321

**Published:** 2024-04-04

**Authors:** Deborah Mojirade Ajayi, Shakeerah Olaide Gbadebo, Osaro Charles Okeaya-Inneh

**Affiliations:** 1 Department of Restorative Dentistry, Faculty of Dentistry, College of Medicine, University of Ibadan, Ibadan, Nigeria,; 2 Department of Restorative Dentistry, University of Calabar, Calabar, Nigeria

**Keywords:** Radiographic evaluation, obturation, endodontic training, root canal treatment

## Abstract

**Introduction:**

the three-dimensional fluid-tight obturation of the root canal system ends the endodontic treatment process and the technical quality of obturation of the root canal (RC) is a determinant of the outcome of the treatment. This final stage of RCT is critical in the outcome of treatment, thus the need to have adequate and quality obturation. The audit of the performance of students in this aspect evaluates performances and identifies where there is a need for improvement. Therefore, we set out to evaluate the quality of root canal obturation performed by undergraduate and postgraduate clinical dental students.

**Methods:**

a cross-sectional study that evaluated the root canal obturation performed by undergraduate and postgraduate students in a teaching hospital, for 1 year. The radiographic evaluation was done by calibrated assessors. The radiographs were viewed under magnifying lenses (x3.5). The adequacy of length and homogeneity of the density of obturation were the outcome variables assessed in all the categories of teeth treated among patients who are 18 years and above, seen during the study period.

**Results:**

eighty-four maxillary and 36 mandibular teeth were root-filled in 97 patients with a mean age of 37.6 years ± 14.7 SD. A good proportion (47.5%) of the root fillings were done by the postgraduate doctors. Most of the canals (69.4%) had acceptable lengths while density was acceptable in only 37.7%. Slightly over half of canals with acceptable length (64 out of 127; 50.4%) were reported in teeth with single canals (p=0.000) likewise with density (28;40.6%). Overall acceptable length and root filling density was 28.9% and there was no statistical significance in the performances of the operators in relation to the length of root filling (p=0.109), and density (p=0.55).

**Conclusion:**

the overall acceptable length and root filling density was 28.9% among both undergraduate and postgraduate students. The adequacy of root canal filling may be dependent on experience, the complexity of the tooth, and the method of instrumentation.

## Introduction

Endodontic treatment often forms an important aspect of the comprehensive rehabilitation of diseased teeth. It acts as a foundation for other treatments such as intra-radicular post placement and tooth preparation for crown and bridge work, by ensuring a total extirpation of the diseased pulp, biomechanical preparation, and complete obturation of the root canal (RC) system; thereby preventing re-infection of the RC system and allowing periapical tissue healing. Being an important aspect of restorative dentistry, it is, therefore, pertinent that both undergraduate and postgraduate clinical students be skilled and competent in every aspect of this treatment modality. Competency in root canal treatment is further necessary to provide adequate treatment, especially in this period with increased demand for RCT borne out of patients´ desire to retain their natural teeth [1-3]. The final component of the endodontic triad is formed by the complete and three-dimensional fluid-tight seal of the root canal system required for a successful endodontic outcome.

The objectives of obturating the root canal system following adequate debridement and preparation are to prevent the passage of microorganisms and fluid along the root canal and to fill the whole canal system, not only to block the apical foramen but also the dentinal tubules and accessory canals. Therefore, the aim of the obturation of the root canal system is to eliminate empty spaces from the coronal to the apical region and preserve the decontamination already achieved by the chemo-mechanical preparation stage of the procedure [4,5]. The quality of root canal obturation is a major determinant that influences the outcome of endodontic treatment. This quality is dependent on the level of obturation; which should be 0-2mm from the radiographic apex, and the density homogeneity of the obturation [6-10].

The various techniques used in obturation, include the age-long cold lateral compaction achieved with gutta-percha (GP) and sealers, a widely used technique considered to be the gold standard for comparing newer techniques. The cold lateral compaction technique is simple, and reliable, with the ability to control the length of fill [11]. However, it may be time-consuming, cumbersome, and associated with the presence of voids between the gutter percha points as well as between the points and the canal walls especially when there is an inadequate clinical skill in this technique [11,12]. Therefore, adequate clinical skill is required to produce a satisfactory obturation with a very high success rate and predictability.

Some studies [13,14] have reported the technical quality of root canal obturation as the main factor that determines the success or prognosis of endodontic treatment, thence, considered the major cause of RCT failure. The success of RCT can be assessed by clinical, radiographic, and histological methods [15]. Evaluation of the technical quality of root canal obturation is generally based on radiographic methods [16,17]. This is a simple, reliable, valid, and objective tool for the quality assessment of root canal obturation. Procedural errors seen on the radiograph that may affect the quality of root canal obturation include root filling ending more than 2mm from the radiographic apex; extrusion of GP and sealant into the periapical tissue; the presence of voids along the canal walls and within the root filling due to inadequate fluid-tight seal [18-20].

The evaluation of the technical quality of root canal obturation is one of the ways of assessing the effectiveness of endodontic training and practice which is done using certain guidelines [5,6]. Many studies have used this to assess the skills and competence of undergraduate students, general practitioners, and even endodontic specialists [7-9]. However, the assessment of postgraduate students has not been presented in many countries. Donnelly *et al*. [21] also pointed out that improvement in clinical performance requires the interaction of factors such as improved or changes in teaching practice, training, and the use of technology. Thus, training and re-training at both undergraduate and postgraduate levels contribute to the improvement of clinical practice. Furthermore, auditing of current teaching and practice is instrumental to improving training and the outcome of treatment being offered.

Therefore, this study set out with the objective of evaluating the quality of the root canal obturation performed by undergraduate and postgraduate clinical dental students. This will help in assessing the effectiveness of undergraduate and postgraduate clinical training in endodontics aimed at developing skilled and competent trainees who will provide quality treatment. Furthermore, the findings from this study may help to formulate a more efficient program that will bring about a significant decrease in the incidence of any undesirable clinical outcomes of endodontically treated teeth that may be associated with poor quality of obturation.

## Methods

**Study design:** this was a cross-sectional observational study.

**Setting:** the study participants consisted of adult patients seen at the Conservative Clinic of the Dental Center of a Tertiary Hospital in Southwest Nigeria. The completely formed permanent teeth of patients aged 18 years and above were included in the study. The operators included 6^th^ year (final year) undergraduate students, house officers (dentists undergoing the one-year compulsory internship post-graduation) as well as postgraduate dental residents/registrars (who are at least 2 years post-graduation and undergoing re-training towards specialization in endodontics). For all the endodontic procedures, the hand instrumentation method of biomechanical preparation of the canal was employed using stainless steel k-files. Working length was determined solely using the radiographic technique and obturation was with the cold lateral compaction technique using gutta percha. The radiographs were taken with E-speed film (PRIMAX RDX-58E soft. Germany) using a paralleling technique with a periapical radiograph film holding device. Poorly processed radiographs and those with superimposed anatomical structures were excluded. Framed radiograph viewers and magnifying lenses (3.5x) were employed for assessment. The results were compared, and a final consensus was reached to collect the appropriate data and the study duly followed the Helsinki Declaration of study on human subjects. The data was prospectively collected within 1 year.

**Participants:** these were patients aged 18 years and above, who had root canal treatment done by the undergraduate and postgraduate students at the Conservative Unit of the Dental Centre of the tertiary teaching Hospital. Only completely formed adult permanent teeth were considered.

**Variables:** the variables considered included the type of tooth, the identified canals, the operator, the length of root filling in each root canal identified, and the density of the root filling. The quality was acceptable if the root filling length was 0-2mm from the radiographic apex and if there was homogenous filling density without internal (between filling) or external voids (between filling and canal wall). Previous acceptable criteria [7-9] were used to evaluate the radiographs in Table 1.

**Table 1:** criteria for evaluation of the technical quality of root canal treatment

**Length of root canal filling**	Adequate	The end of root canal filling is either at or ≤ 2 mm from the radiographic apex
	Inadequate	Root canal filling is either too short (more than 2 mm from the radiographic apex) or extends beyond the radiographic apex
**Density of root canal filling**	Adequate	No voids are visible within the root canal filling or between the filling and the canal walls
	Inadequate	Voids are visible within the root canal filling or between the filling and the canal walls

**Data source/measurement:** radiographs of all root canal treatments were used and evaluated for the length and density of compaction of the root filling. The quality of the root canal treatment was determined by the length of root fillings in relation to the radiographic apex and the density of obturation according to the presence or absence of voids. Previous acceptable criteria [7-9] were used to evaluate the radiographs. The evaluation of the radiographs was done by three previously calibrated independent assessors who were blinded to the individuals who performed the RCT. Calibration was done with 5 radiographs each for the different types of teeth viewed under the magnifying lenses for reliability and confidence in assessment. The adequate length was acceptable if it was 0-2mm from the radiographic apex of the tooth and reported as 'acceptable' and if not as 'not acceptable'. The paralleling technique is a widely used method of intra-oral radiography [22] and was the method employed in this study. The canals that were superimposed/overlapped on the radiograph of the molars, but recorded clinically were referred to as ´merged canals´ in this study.

**Study size:** convenience sampling was employed. All post-obturation periapical radiographs of root canal treatment (RCT) performed by undergraduate and postgraduate students were prospectively collected within 1 year.

**Quantitative:** the quantitative data were the age, the number of teeth and identified canals, and the frequencies including the mean age were presented.

**Statistical methods:** data collected were entered, cleaned, and analyzed using Statistical software for Social Sciences (IBM SPSS version 23). Descriptive statistics were used to analyze the frequency in relation to age, gender of participants, the operators, distribution of teeth treated, and the length and density of teeth treated. These are presented in tables and charts. The adequacy of root canal length and density were compared among the operators and the type of canal using Chi-square at 5% significance.

**Ethical approval:** the conduct of the research was consistent with the declaration of Helsinki on ethical principles for research involving human subjects with ethical approval sought and obtained from the University of Ibadan/University College Hospital (UI/UCH) ethics and review committee board (UI/EC/24/0314). Informed consent was also signed by the participants, and other ethical considerations associated with data management such as confidentiality, were maintained.

## Results

**Figure 1:**
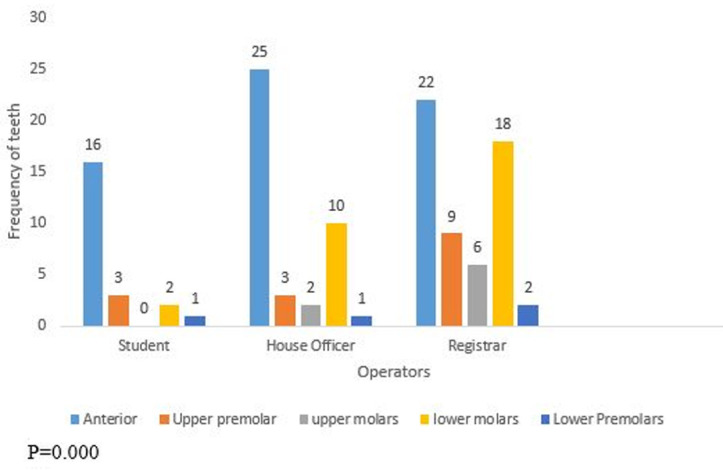
distribution of teeth as treated by operators

**Participants:** one hundred and twenty teeth (84 maxillary and 36 mandibular) were root-filled in 97 patients (45 males, 52 females) with a mean age of 37.6±14.7 years. A greater percentage (47.5%) of the teeth were treated by registrars (postgraduate resident doctors) followed by house officers (34.2%). About 40% of the anterior teeth were treated by house officers, most of the lower molars were treated by the registrars while none of the maxillary molar teeth, though few, were treated by the undergraduate students (Figure 1).

**Descriptive data:** a total of 70% of the root-filled teeth were in the maxilla, with the lowest proportion (14.2%) found in the mandibular right quadrant and the highest (40%) in the maxillary right quadrant. Over half (63, 52.5%) of the treated teeth were single-rooted anterior teeth (Table 2). Table 3 shows that the total of the minimum expected number of canals in the 120 root-filled teeth was 209, out of which 183 (87.6%) were identified radiographically. The mandibular molars had the highest proportion (22 of 30; 73.3%) of merged canals, with the two canals in the mesial roots appearing merged into single canals termed mesial canals. Furthermore, all the single-rooted anterior teeth had their canals completely identified and it was also possible to identify all the canals expected in the majority (7 out of 8, 87.5%) of the maxillary molars. The total expected number in the maxillary second premolar was put at two per tooth but could also be one per tooth depending on the race studied.

**Table 2:** distribution of root-treated teeth

Maxillary teeth																		
Tooth type	18	17	16	15	14	13	12	11	Total N (%)	21	22	23	24	25	26	27	28	Total N (%)
Number treated	0	2	0	0	7	3	6	30	**48(40.0)**	16	6	0	6	2	5	1	0	**36 (30.0)**
**Mandibular teeth**																		
Tooth type	48	47	46	45	44	43	42	41	Total N (%)	31	32	33	34	35	36	37	38	Total N (%)
Number treated	1	7	5	2	0	0	0	2	**17 (14.2)**	0	0	0	0	2	3	12	2	**19 (15.8)**

**Table 3:** number of canals identified radiographically

Tooth type	Total n^o^ of teeth	Minimum expected number of canals	Total number of canals identified									No of radiographically unidentified canals
	N (%)		P	B	D	DB	ML	MB	S	ME	T; N (%)	N (%)
Anterior	63 (100)	63	NA	NA	NA	NA	NA	NA	63	NA	63 (100)	0 (0)
Upper 1st premolar	13 (100)	26	10	10	NA	NA	NA	NA	3	-	23 (88.5)	3 (11.5)
Upper 2nd premolar	2 (100)	4	0	0	NA	NA	NA	NA	2	-	2 (50)	2 (50)
Upper molars	8 (100)	24	8	0	0	8	NA	7	0	0	23 (95.8)	1 (4.2)
Lower molars	30 (100)	90	NA	NA	30	NA	8	8	0	22	68 (75.6)	22 (24.4)
Lower premolars	4 (100)	4	0	0	0	0	0	0	4	0	4 (100)	0 (0)

NA= Not Applicable, P= Palatal, B= Buccal, D= Distal, DB= Distobuccal, ML= Mesiolingual, MB= Mesiobuccal, S= Single canal, ME= Merged canals (in a tooth where more than one canal is expected), T= total number of canals seen radiographically

**Main result/outcome data:** the length of the canal on the radiograph was considered acceptable when the length of obturation was between 0-2mm short or beyond the radiographic apex, while anything greater than 2mm was taken as an unacceptable root-filling length. The majority of the canals 69.4% had acceptable length and slightly over half of them (64 out of 127; 50.4%) of these were reported in teeth with single canals as compared to other teeth, (p=0.000). The distal canal of the mandibular molar had the highest proportion (12 of 56; 21.4%) of unacceptable canals, followed closely by the mesial canals of the same tooth type (11 of 56; 19.6%) (Table 4).

**Table 4:** the quality of root canal filling lengths in various canals

Type of canal	Length		
	Acceptable; N (%)	Not acceptable; N (%)	Total
Single	64 (35.0)	8 (4.4)	72
Distal canal	18 (9.8)	12 (6.6)	30
Mesio-buccal canal	7 (3.8)	8 (4.4)	15
Mesial (merged MB/ML canal lower)	11 (6.0)	11 (6.0)	22
Palatal	14 (7.7)	4 (2.2)	18
Disto-buccal	5 (2.7)	3 (1.6)	8
Buccal	5 (2.7)	5 (2.7)	10
Mesiolingual	3 (1.6)	5 (2.7)	8
Total	127 (69.4)	56 (30.6)	183

P<0.001; ML= Mesiolingual; MB= Mesiobuccal

The acceptability or otherwise of the density of root filling is shown in Table 5. The internal (within root filling) and external (between root filling and canal walls) voids as detected in various canals are presented in the table. The density of root filling was acceptable in only 37.7% of the 183 canals identified radiographically. The single canals had the highest number (28;40.6%) among the canals with acceptable density. The majority (98; 86.0%) of the total root-filled teeth that were deemed unacceptable had internal voids. While none of the identified mesiolingual canals had acceptable density, only 3 out of 15 (20.0%) identified MB canals had acceptable density. In Table 6, the performance of different categories of operators as related to the radiographic quality of root fillings is presented. With regards to the length of root filling, almost all (23/26, 88.5%) of the canals filled by the students were acceptable, followed by those done by the registrars (66/97, 68.0%). Conversely, the registrars had the highest proportion (40.2%) of canals with acceptable density, followed by the house officers (35%) and then the students (34.5%). None of the teeth filled by the students had both internal and external voids combined. Overall acceptable length and root filling density was 28.9% and there was no statistical significance in the performances of the operators in relation to the length of root filling (p=0.109), and density (p=0.55)

**Table 5:** density of root filling in the different root canals

Type of canal	Acceptable; N (%)	Not acceptable (internal voids); N (%)	Not acceptable (external voids); N (%)	Not acceptable (internal + external voids); N (%)	Total; N (%)
Single	28 (15.3)	38 (20.8)	3 (1.6)	3 (1.6)	72 (39.3)
Distal canal	14 (7.7)	15 (8.2)	1 (0.6)	0 (0)	30 (16.4)
Mesiobuccal canal	3 (1.6)	11 (6.0)	0 (0)	1 (0.6)	15 (8.2)
Mesial	10 (5.5)	9 (4.9)	3 (1.6)	0 (0)	22 (12.0)
Palatal	9 (4.9)	7 (3.8)	1 (0.6)	1 (0.6)	18 (9.8)
Distobuccal	2 (1.1)	6 (3.3)	0 (0)	0 (0)	8 (4.4)
Buccal	3 (1.6)	6 (3.3)	1 (0.6)	0 (0)	10 (5.5)
Mesiolingual	0 (0)	6 (3.3)	2 (1.1)	0 (0)	8 (4.4)
Total	69 (37.7)	98 (53.6)	11 (6.0)	5 (2.7)	183 (100)

**Table 6:** acceptability of radiographic quality of root canal fillings according to operators

Variables	Operators			Total	P-value
Length of root filling	Students	House officers	Registrars		
Acceptable	23	38	66	127	
Not acceptable	3	22	31	56	0.109
**Total**	**26**	**60**	**97**	**183**	
**Density of root filling**					
Acceptable	9	21	39	69	
Not acceptable (internal voids)	15	29	54	98	0.055
Not acceptable (external voids)	2	6	3	11	
Not acceptable (internal+external voids)	0	3	2	5	
**Total**	**26**	**60**	**97**	**183**	

In Table 6, the performance of different categories of operators as related to the radiographic quality of root fillings is presented. With regards to the length of root filling, almost all (23/26, 88.5%) of the canals filled by the students were acceptable, followed by those done by the registrars (66/97, 68.0%). Conversely, the registrars had the highest proportion (40.2%) of canals with acceptable density, followed by the house officers (35%) and then the students (34.5%). None of the teeth filled by the students had both internal and external voids combined. Overall acceptable length and root filling density was 28.9% and there was no statistical significance in the performances of the operators in relation to the length of root filling (p=0.109), and density (p=0.55).

## Discussion

The demand for root canal treatment (RCT) is increasing in Nigeria [23,24], therefore, the quality of the treatment must follow the prescribed international standard which will lead to healing of the pulpal pathology and satisfactory patient´s condition. This study looked at the adequacy and quality of root canal filling as performed by final-year undergraduate students and different cadres of doctors on different types of teeth.

Most of the teeth treated in this study were the maxillary incisors. This follows the report of many studies [25-27]. The maxillary anterior teeth (the central and lateral incisors) are the major teeth that undergo RCT basically due to their position which makes them liable to pulpal disease secondary to trauma. Also, the unaesthetic tooth discoloration that may occur following the trauma to anterior teeth is another reason that may prompt the patient to seek the treatment, RCT. Although Adebayo *et al*. [27] observed from their study that more teeth were endodontically treated in the mandibular region than the maxillary, the majority of the teeth in the maxillary were still the anterior (central, lateral incisors, and canine) teeth.

The teeth treated by registrars (postgraduate resident doctors) were more (47.5%) followed by the House officers while the students had the least. Also, most of the posterior teeth both maxillary and mandibular were treated by the registrars. This corroborates a previous study [28] that reported less root canal treatment especially posterior teeth being assigned to undergraduate students at a teaching hospital setting. This may be due to less confidence and competence being shown by undergraduate students in handling such tooth types. Therefore, more training especially laboratory endodontic training needs to be included in the undergraduate curriculum to improve clinical skills and boost the confidence of this set of trainees as documented by some researchers [9,29]. In addition, the registrars make up the bulk of doctors in a teaching hospital setting and thus, may be involved more in the treatment of patients especially because they also have requirements to fulfill to be qualified for the postgraduate examinations of different colleges.

The present study assessed the filling of each canal after identification on a periapical radiograph. However, the identification of some of the canals showed the merging/overlapping of more than 70% of the mesial canals into single canals. Identification of root canals is done conventionally by use of 2-dimensional plain periapical radiographs, and the paralleling technique used in this study is the most prescribed due to its advantages [22,30]. However, a shortcoming of the technique is the superimposition/overlapping of roots, canals, and proximal area if the horizontal angulation of the cone is not well placed on the film sensor [31]. However, this can be avoided by taking the radiographs with a film holder or at different angulations with at least two radiographs [32]. Also, the use of advanced radiology such as CBCT has been recommended to give better results and less radiation [32]. However, the cost and availability may be the barrier.

Most of the canals (69.4%) had adequate and acceptable lengths, and this is similar to the report by Er *et al*. [33] and within the range of 63.5%-70% recorded by Mustafa [34]. This finding is also close to a previous similar study by Adebayo *et al*. [27] who reported 71% adequate length in their study conducted also in Nigeria. However, it is higher than the 61% presented by Barrieshi-Nusair *et al*. [8] and Arigbede *et al*. [25]. There is, nevertheless, a difference in the study participants. The participants in the present study were a mix of different cadres of doctors and final-year undergraduate students, while Adebayo *et al*. [27] considered different cadres of doctors categorized based on years of experience, and Er *et al*. [33] and Mustafa [34] studied the quality of obturation performed by undergraduate students only. The comparison of postgraduate and undergraduate students (who were final year undergraduates) in our study may give insight into how prepared the undergraduates are and how much more skill has been acquired by the postgraduate students who are mostly doctors with >5 years post-undergraduate training/post qualification.

The majority of the canals with adequate length were anterior teeth. The relationship between the adequate length of obturation among canals in the anterior and posterior teeth was found to be statistically significant (p=0.000). Barrieshi-Nusair *et al*. [8] reported the same findings in their study. This also corroborates the result from Mustafa´s study [34] that reported higher adequacy in obturation length in anterior teeth which mainly have single canals that are usually straight. The present study considered root canals individually such as was done by Donelly *et al*. [21] unlike some other studies [25,27] that considered teeth. The peculiarity of each canal in every tooth should be considered when assessing the quality of the length of root filling. The morphology of the root canal particularly the degree of curvature may affect this variable. The complexity in canal length as regards posterior teeth was also shown by Mustafa [33] as the majority of the posterior teeth treated by final year (5^th^ year) students had lower acceptable adequacy in root canal length filling.

Almost all of the canals filled by the students were acceptable in length, followed by those done by the registrars (postgraduate students). This follows the reports of some studies [3,1,34,35] that have reported acceptable root canal length filling by undergraduate students. The majority of the teeth treated by the undergraduate students were anterior teeth with single canals, unlike the other cadres of doctors who treated posterior teeth. It also follows the report that the acceptability of the quality of the root filling decreases as one moves towards the posterior teeth [36,37]. This is further explained by the complexity of the anatomy of posterior teeth as well as the poor accessibility to such teeth at times, unlike the anterior single-rooted teeth which usually present with straight roots with good taper and less degree of curvature (if any at all) and aberration.

The total number of teeth with acceptable density (homogeneity) was low in our study (37.2%) compared to that reported by Pietrzycka *et al*. [3,8] and Arigbede *et al*. [25] that found the majority of the teeth (97.69% and 72.1% respectively) had adequate homogeneity. However, our result is higher than that of Kielbassa *et al*. [39] which reported 70.4% non-homogeneity (29.6% homogeneity), and Hayes *et al*. [40]. Internal voids were responsible for the majority of the root-filled teeth that were deemed unacceptable in homogeneity in this study. The combination of hand instrumentation and cold lateral compaction technique of obturation employed in this study is used in many other studies [25,27,34]. This may show voids when the gutta percha cones are not well compacted unlike in single cone technique with rotary biomechanical instrumentation. It may, therefore, be necessary to compare the quality of root filling done with rotary instrumentation and hand instrumentation among all the cadres of doctors to see if there will be a difference as regards root filling adequacy and acceptability.

The registrars had the highest proportion of canals with acceptable density, followed by the house officers and then the students. This is converse to the study by Adebayo *et al*. [27] where the junior doctors (0-5 years post-graduation) had more teeth with good quality root filling than the senior dentists (> 5 years post-graduation), though this was not statistically significant (p=0.34). The registrars in this study, however, may fall into the category of ≥ 5 years post-qualification. Also, the study center is a teaching hospital where more clinical knowledge on endodontics would have been gained by the post-graduate students compared to the final year undergraduates, and the fact that the students see fewer patients compared to the post-graduate doctors as reported in a previous study [28].

The total percentage acceptable density and length of 28.9% in our study was similar to the report of Román-Richon *et al*. [41] but lower than that reported by Pietrzycka *et al*. [38], Moradi *et al*. [42] Adebayo *et al*. [27] and higher than that reported by Hayes *et al*. [41]. The differences shown here may be due to instrumentations as all the RCT reviewed in this study were done with hand instrumentation, unlike other studies [38,41] where rotary instrumentation was also used and compared with manual. However, the cold lateral compaction technique was used just like in many other studies that employed hand instrumentation [27,38,4,1]. Furthermore, Ribeiro *et al*. [36] showed the very low quality of root filling among undergraduates using hand instrumentation while another study [41] found better quality of the root filling in RCT performed with rotary instrumentation compared to manual. This, therefore, shows the need to introduce and adopt advances in endodontic treatment for a better treatment outcome and an improvement in endodontic training.

Furthermore, the combination of different cadres of doctors (both undergraduate and postgraduate) might be responsible for this result. However, Pietrzycka *et al*. [38] also considered doctors with different levels of experience and reported a better overall acceptable adequate root filling. Interestingly, they reported that the Endodontists had the lowest adequate root canal length filling of 74.48% (due to overfilling of canals) next to 4^th^ year undergraduate students, however, they had the second highest adequate canal density. Thus, experience and adequate exposure may matter for adequate endodontic treatment. Nevertheless, it may be necessary to assess the different cadres of doctors separately. In addition, this result calls for more clinical training and exposure of undergraduate as well as postgraduate students to acquire adequate skills and competencies in basic endodontic procedures for a better treatment outcome.

The quality of root filling is directly related to the better outcome of root canal treatment. This study, however, did not relate the findings in the quality of the root filling with the outcome of the treatment as the follow-up of the treatment was not part of the scope of this research. Therefore, further research relating to the treatment outcome of cases with inadequacies in the length and density qualities of root filling and coronal restoration may be necessary as a follow-up to this study.

The strength of this study is the assessment of individual root canals instead of the teeth as well as the comparison of different cadres of doctors and undergraduate students. The sole use of radiographic assessment in this study, without the inclusion of the clinical outcome of the root canal post-treatment, is however, a limitation but clinical assessment was not in the scope of this study.

## Conclusion

The adequacy of root canal filling may be dependent on experience, the complexity of the tooth, and the method of instrumentation. It may, therefore, be necessary to introduce and expose doctors and students (undergraduate) to more training, and advances that are, in the field of endodontics for better treatment outcomes.

### What is known about this topic

- *Adequacy of root canal obturation can impact positively on the overall success of the treatment*.

### What this study adds

- *Evaluation of students’ performances both at undergraduate and postgraduate levels can reveal the muddiest point of the treatment and give room for improvement;*
- *The importance of training and re-training students on this significant aspect of the specialty of endodontics is crucial for their optimal performance*.
